# CD71^+^ Erythroid Cells in Human Neonates Exhibit Immunosuppressive Properties and Compromise Immune Response Against Systemic Infection in Neonatal Mice

**DOI:** 10.3389/fimmu.2020.597433

**Published:** 2020-11-24

**Authors:** Shokrollah Elahi, Marco Antonio Vega-López, Vladimir Herman-Miguel, Carmen Ramírez-Estudillo, Javier Mancilla-Ramírez, Bruce Motyka, Lori West, Olaide Oyegbami

**Affiliations:** ^1^ School of Dentistry, University of Alberta, Edmonton, AB, Canada; ^2^ Department of Medical Microbiology and Immunology, University of Alberta, Edmonton AB, Canada; ^3^ Department of Oncology, University of Alberta, Edmonton, AB, Canada; ^4^ Faculty of Medicine and Dentistry, Li Ka Shing Institute of Virology, University of Alberta, Edmonton, AB, Canada; ^5^ Dept. Infectómica y Patogénesis Molecular, Centro de Investigación y de Estudios Avanzados del IPN, Mexico City, Mexico; ^6^ Escuela Superior deMedicina, Instituto Politecnico Nacional, Hospital de la Mujer, Secretaria de Salud, Mexico City, Mexico; ^7^ Alberta Transplant Institute and the Canadian Donation and Transplantation Research Program, Edmonton, AB, Canada; ^8^ Department of Pediatrics, University of Alberta, Edmonton, AB, Canada; ^9^ Department of Surgery, University of Alberta, Edmonton, AB, Canada; ^10^ Department of Laboratory Medicine & Pathology, University of Alberta, Edmonton, AB, Canada

**Keywords:** CD71^+^ erythroid cells, newborns, neonatal infections, immunosuppression, COVID-19

## Abstract

Newborns are highly susceptible to infectious diseases. The underlying mechanism of neonatal infection susceptibility has generally been related to their under-developed immune system. Nevertheless, this notion has recently been challenged by the discovery of the physiological abundance of immunosuppressive erythroid precursors **C**D71^+^ **e**rythroid **c**ell**s** (CECs) in newborn mice and human cord blood. Here, as proof of concept, we show that these cells are also abundant in the peripheral blood of human newborns. Although their frequency appears to be more variable compared to their counterparts in mice, they rapidly decline by 4 weeks of age. However, their proportion remains significantly higher in infants up to six months of age compared to older infants. We found CD45 expressing CECs, as erythroid progenitors, were the prominent source of reactive oxygen species (ROS) production in both humans and mice. Interestingly, a higher proportion of CD45^+^CECs was observed in the spleen versus bone marrow of neonatal mice, which was associated with a higher ROS production by splenic CECs compared to their siblings in the bone marrow. CECs from human newborns suppressed cytokine production by CD14 monocytes and T cells, which was partially abrogated by apocynin *in vitro*. Moreover, the depletion of CECs in neonatal mice increased the number of activated effector immune cells in their spleen and liver, which rendered them more resistant to *Listeria monocytogenes* infection. This was evident by a significant reduction in the bacteria load in the spleen, liver and brain of treated-mice compared to the control group, which enhanced their survival rate. Our finding highlights the immunoregulatory processes mediated by CECs in newborns. Thus, such tightly regulated immune system in newborns/infants may explain one potential mechanism for the asymptomatic or mild COVID-19 infection in this population.

## Introduction

Infectious disease is still a major global cause of childhood mortality (1, 2). Neonates are highly susceptible to a variety of infectious agents, which are often fatal and causing ~ 700,000 deaths per year (1–3). While infant mortality is < 5 per 1,000 live births in developed states, this rate is often > 30 times higher in resource-limited countries (4). Even in resource-rich countries, infections in young infants incur an enormous burden; approximately each infectious disease hospitalization for every 14 infants in the U.S. results in an annual cost of ~$700 Million (5). Of the serious infections in infants, sepsis, and meningitis carry the highest morbidity and mortality rates (6, 7). Considering the magnitude of this global health problem, even moderate efficient interventions can save millions of lives and billions of dollars. Enhancing the neonatal immune responses against pathogens through immune modulation/vaccination appears to be an attractive approach. However, this will not be possible unless we gain a better and deeper insight into developmental changes occurring in the neonatal immune system at the cellular and molecular levels.

There are clear qualitative and quantitative differences in both innate and adaptive immune systems between adults and neonates, which at least partially can explain the increased susceptibility to infection (8). The fetal immune system is biased towards an anti-inflammatory, T helper 2 (Th2) response, as is that of the pregnant woman. The fetus is antigenically different from its mother and could be compared to the immunological mismatch that can occur during transplantation with the risk of rejection (9–11). As such, the immune response during pregnancy appears to have evolved to prevent potentially damaging inflammation that otherwise may result in abortion or preterm delivery (12).

As beneficial as the tolerogenic state might be *in utero*, growing evidence suggests that this may predispose the newborn to severe infections and impairs their immune responses to vaccinations in postnatal life.

Another important event to consider is the sudden removal of the fetus from a highly privileged and protected environment that is aquatic, warm, and almost sterile, to a new, cold and dry environment full of pathogenic and/or non-pathogenic microorganisms and other antigens. The newborn faces many challenges in this new environment while growth and development are her/his top priority. Therefore, a highly dynamic and tolerant immune system is necessary for the newborn’s survival. Initially, this tolerogenic phenomenon was related to the immune system immaturity in newborns (9, 12). However, this concept has recently been challenged and replaced by a novel concept (13). Neonatal infection susceptibility results from the temporal presence of physiologically enriched immunosuppressive erythroid precursors (13, 14). These erythroid precursors co-express the transferrin receptor CD71 and erythroid marker TER119 in mice, and CD71 and CD235a in humans (13, 14). CD71^+^ erythroid cells (CECs) are abundant in the spleen of neonatal mice (13–15), placenta tissues and expand during pregnancy in the peripheral blood/spleen of allogeneic mice (16). Similarly, they are enriched in the human cord blood, placenta tissues, the peripheral blood during pregnancy (17–20) and in the liver of human neonates (21). CECs exhibit immunosuppressive functions regardless of their anatomical location (22). More importantly, CECs deplete L-arginine by the expression of arginase-II and subsequently impair immune activation in antigen-presenting cells and T cells *in vitro* (13, 14). In agreement with our observations, the expansion of immunosuppressive CECs in the spleen of mice in a tumor model of melanoma has also been reported that CECs inhibit antigen-specific CD8^+^ T cell responses *via* the production of reactive oxygen species (ROS) (23). CECs from the mice placental tissue co-express PDL-1/PDL-2 and *via* interaction of these ligands with PD-1 on T cells suppress IFN-γ production *in vitro* (16). These observations demonstrate that CECs are a heterogeneous population of erythroid progenitors and precursors (22) and depending on their microenvironmental localization may utilize different mechanisms to suppress or modulate immune responses (8, 24). Moreover, splenic CECs express V-domain Ig Suppressor of T Cell Activation (VISTA) (25), a newly discovered inhibitory receptor and a transmembrane immunoglobulin superfamily also known as (PD-1H, vsir) (26–28). VISTA+CECs are the major source of TGF-β and *via* this cytokine promote the induction of regulatory T cells (Tregs), while VISTA^−^CECs or CECs from VISTA-KO mice failed to promote the induction of Tregs (25). Overall, these pieces of evidence demonstrate a wide range of immunological properties for these less appreciated cells.

Although the presence of reticulocytes from the hematological perspective in human infancy has been reported (29), their biological properties have never been investigated. Therefore, in this study we investigated the frequency and functionality of CECs in the peripheral blood of human newborns/children from 1-day to 5-year and compared them with their counterparts in the neonatal mice. This study, for the very first time, provides a novel insight into the immunomodulatory capabilities of CECs in human newborns. Besides, we utilized a neonatal mouse model of *Listeria monocytogenes* (L.m) infection, as a significant cause of meningitis (30) in infants, to evaluate the role of CECs *in vivo*.

## Methods

### Human Sample Collection and Processing

Blood was collected from healthy adult volunteers, and cord blood from full-term deliveries at the Grey Nuns Community Hospital, Edmonton. Thereafter, peripheral blood mononuclear cells (PBMCs) or cord blood mononuclear cells (CBMCs) were isolated over Ficoll-Hypaque gradients. For CD71^+^ cell or mock depletion, cord blood samples were stained using anti-CD71 or isotype control biotin-conjugated antibody and fractioned using streptavidin linked magnetic beads (Miltenyi Biotec) according to our previous reports (13, 14, 17, 31). The appropriate Institutional Human Review Ethics Boards at the University of Alberta approved human studies with the ethics # Pro0046080 and Pro00063463. All study participants gave written informed consent to participate in this study. Studies related to human newborns were mainly performed in Mexico. The Ethics Committee of the Hospital de la Mujer (Women´s Hospital), the Mexican Ministry of Health approved the study (Reg. HM-INV/2018:02.09). In addition, some neonatal blood specimens were collected at the University of Alberta Hospital from infants who had elective operations. The appropriate Institutional Human Review Ethics Boards at the University of Alberta approved such studies (ethics # Pro00001408). Parents gave written informed consent form to participate in the neonatal related studies in Mexico and Canada.

### Animals

Male and female BALB/c mice were purchased from the Charles River Institute. BALB/c mice were bred together, and pregnant mice were checked twice daily to establish birth timing. All animals were maintained and bred under pathogen free conditions within the animal care facility at the University of Alberta. This study was carried out in strict accordance with the recommendations in the Guide for the Care and Use of Laboratory Animals of the Canadian Council for Animal Care with animal ethics # AUP00001021.

### The Experimental Model of Bacterial Infection

*Listeria monocytogenes* (L.m) was used as a disease model in newborn mice but the sex was not identifiable. For *in vivo* depletion of CECs, 80 μg purified anti-CD71 antibody (clone 8D3) was administered intraperitoneally (i.p.) at 4 and 5 days of age according to previous reports (13, 14). The same amount of IgG2a isotype control was injected i.p. into different neonatal mice to serve as controls. On day 6, mice were orally fed with ~10^7^ colony forming units (CFUs) of L. m in 10 μl PBS. Two days later animals were euthanized for harvesting the spleen, liver, and brain. Bacterial counts were assessed by plating serial dilutions of the homogenates onto Luria broth (LB) plates and incubation at 37°C for 1–2 days. In parallel, some neonatal mice were fed with PBS to serve as controls. The similar infection approach was used for the survival study. Neonatal mice were monitored twice a day to record any mortality and kept up to 2 weeks post-infection.

### Antibodies and Flow Cytometry

Fluorophore or biotin-conjugated antibodies with specificity to mouse cell surface antigens and cytokines were purchased from BD Biosciences or Thermo Fisher Scientific. Specifically, the following antibodies were used: anti-CD71 (R17217 and C2F2), anti-Ter119 (TER-119), anti-CD45 (30-F11), anti-VISTA (MIH64), anti-CD11b (M1/70), anti-CD11c (N418), anti-B220 (RA3-6B2), anti-CD40 (1C10), anti-CD80 (16-10A1), anti-CD86 (GL1), anti-CD3 (145-2C11), anti-CD4 (GK1.5), and anti-CD8a (53–6.7) for mice, For human studies, the following fluorophore or biotin-conjugated antibodies with specificity to surface markers or cytokines were used: anti-CD3 (HIT3a), anti-CD4 (RPA-T4), anti-CD8 (RPA-T8), anti-CD45 (H-130 or 2D1), anti-VISTA (B7H5DS8) and anti-IFN-γ (4S.B3), anti-CD71 (MA712), and anti-CD235A (HIR2). ROS staining (Sigma) was performed by flow cytometry per the manufacturer’s protocols and our previous reports (17, 31). Live/dead fixable dead cell stains (ThermoFisher) were used to exclude dead cells in flow cytometry. Paraformaldehyde fixed cells were acquired by flow cytometry using a LSRFORTESSA flow cytometer (BD) and analyzed with FlowJo software.

### Co-Culture and Stimulation

For *in vitro* intracellular cytokine staining, neonatal PBMCs were cultured and stimulated with anti-CD3/CD28 in RPMI media supplemented with 10% FBS for 24 h in the presence or absence of CECs according to our previous report (15). For co-culture, a fixed number (1 x 10^5^) of PBMCs were seeded into 96 well round bottom plates individually or together with neonatal CECs at 1:1 ratio, Brefeldin A (10 μg/ml) was added 6 h prior to analysis. In some experiments, apocynin (1–2 mM) was added at the time of stimulation to abrogate the effects of ROS *in vitro* (31). For mice studies, splenocytes were harvested, and single-cell suspensions were made by grinding between sterile frosted glass slides in RBC lysis buffer and filtering through nylon mesh. Splenocytes were washed by centrifugation and used for subsequent *in vitro* studies.

### Statistical Analysis

Statistical comparison between various groups was performed by the t-test using PRISM, Graph Pad software. Also, differences were evaluated using One-Way ANOVA followed by Tukey’s test for multiple comparisons. Results are expressed as mean± SEM. P-value <0.05 was considered as statistically significant.

## Results

### CECs Are Physiologically Abundant in Human Newborns but Their Frequency Declines With Age

**Figure 1:**
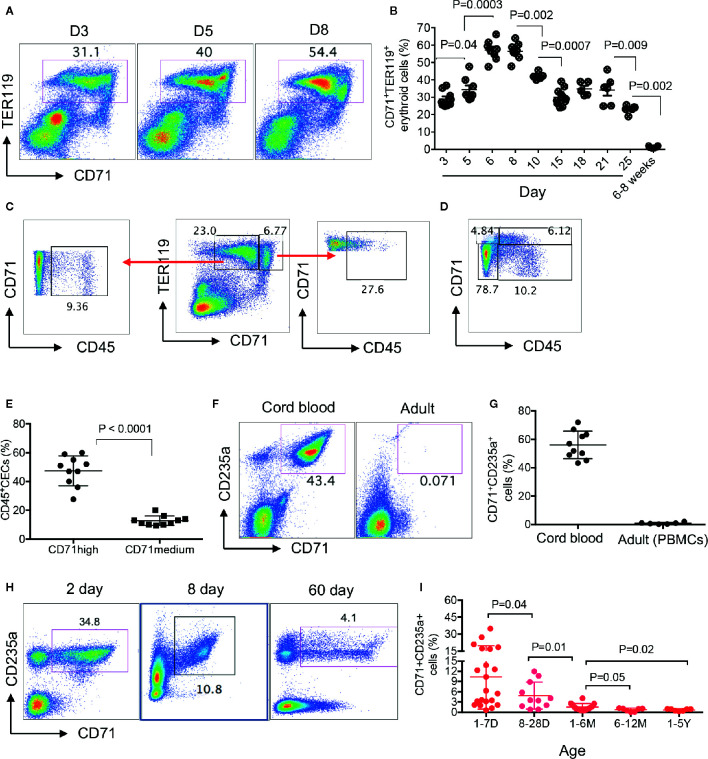
Physiological abundance of CECs in newborns. **(A)** Representative flow cytometry plots of frequency of CECs in the spleen of days 3, 5, and 8 mice. **(B)** Cumulative data showing percentages of CECs in spleens of day-3 to adult (6–8 weeks) mice. **(C, D)** Representative plots showing percentages of CD45^+^ in TER119^+^CD71medium and TER119^+^CD71high CECs. **(E)** Cumulative data showing percentages of CD45+ subpopulation of CECs in TER119^+^CD71medium and TER119^+^CD71high CECs in the spleen of neonatal mice. **(F)** Representative plots of frequency of CECs in the cord blood or the peripheral blood of human adults. **(G)** Cumulative data showing the percentages of CECs in the cord blood versus the peripheral blood of adult humans. **(H)** Representative plots of frequency of CECs in the peripheral blood of human newborns (days 2 and 8 and 2 months). **(I)** Cumulative data showing the frequency of CECs in the peripheral blood of human infants/children from day 1 to 5 years of age. Each point represents data from an individual mouse or human subject (adult, cord blood, or neonatal blood). Bar, mean ± one standard error.

We have previously shown that CECs were physiologically enriched in the spleen of 6–28 days old neonatal mice (13, 14). Here, we quantified the frequency of CECs in the spleen of day-3 to adult mice and found that the frequency of CECs was significantly lower in day-3 and day-5 compared to day-6. These cells enriched to their maximum levels at days 6 to 9 before beginning to gradually disappear by 4 weeks of age (**Figures 1A, B**, **Supplementary Figure 1A**). When analyzing CECs in the spleen of neonatal mice, we noticed two subpopulations of CECs defined as CD71midTER119^+^ and CD71highTER119^+^ cells (**Figure 1C**). Our further investigations indicated that CD71highTER119^+^ subpopulations were enriched with CD45^+^CECs (**Figures 1D, E**). Although erythrocytes do not express CD45 (32), they are generated from CD45+ hematopoietic stem cells (HSC) and downstream erythroid progenitors through cytokine signaling such as erythropoietin (EPO) and stem cell factor (33). In agreement with previous reports, we found that the human cord blood was enriched with CECs while they were almost absent or at very low frequency in the peripheral blood of human adults (**Figures 1F, G**). For the very first time, to our knowledge, we found that CECs were abundant in the peripheral blood of human neonates prior to day-28. The frequency of CECs was significantly higher in the peripheral blood of 1–7 day old compared to 8- to 28-day-old neonates but significantly declined thereafter (**Figures 1H, I**, **Supplementary Figure 1B**). Despite their substantial reduction by 4 weeks, we found their frequency was still significantly higher in age group (1–6M) compared to those older groups (**Figure 1I**). These observations indicate that human newborns similar to mice newborns are physiologically enriched with CECs at the early stage of life.

### CECs Are Heterogeneous and CD45^+^CECs Are a Greater Source of ROS Than CD45^−^ CECs

**Figure 2:**
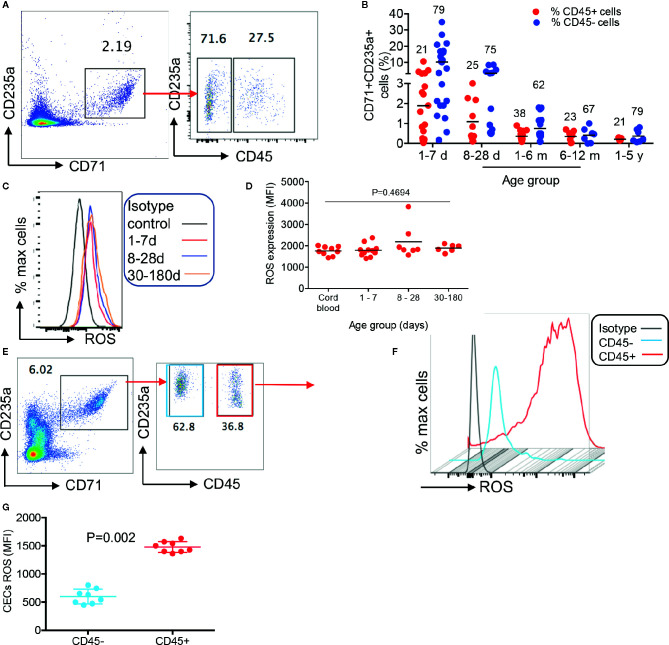
Differential expression of ROS by CD45^+^ versus CD45^−^CECs in humans. **(A)** Representative plot showing differential expression of CD45 in CECs from a human infant. **(B)** Cumulative data showing percentages of CD45^+^ and CD45^−^ CECs in human infants/children from day-1 to 5 years of age. The numbers on red and blue symbols showing % CD45^+^ or CD45^−^CECs. **(C)** Representative histogram of ROS expression in CECs, and **(D)** cumulative data showing mean fluorescence intensity (MFI) of ROS in CECs from human cord blood and neonatal blood at shown ages. **(E)** Representative plots showing frequency of CD45^+^ versus CD45^−^ CECs in the peripheral blood of an 8-day-old human newborn. **(F)** Representative histogram of ROS expression in CD45^+^ versus CD45^−^CECs in an 8-day-old human newborn. **(G)** Cumulative data showing mean fluorescence intensity (MFI) of ROS in CD45^+^/CD45^−^CECs of human newborns.

**Figure 3:**
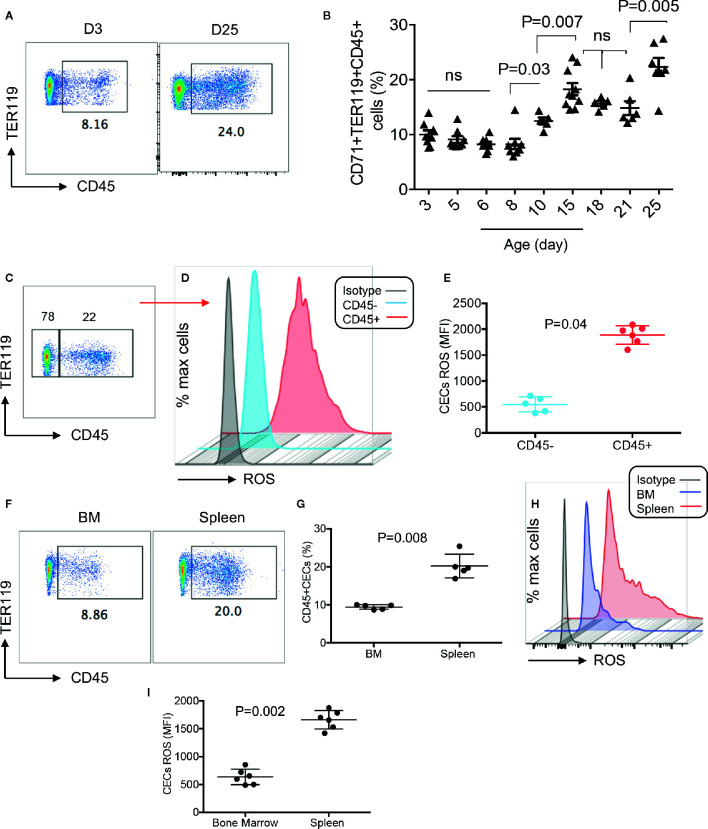
Differential expression of ROS by CD45+ and CD45− CECs in mice. **(A)** Representative plots showing percentages of CD45+ subpopulation among mice splenic CECs at day-3 and day-25. **(B)** Cumulative data showing percentages of CD45+ CECs among splenic mice CECs from day 3 to day 25. **(C)** Representative plot of CD45 expressing CECs in splenic CECs of a 15-day-old mice. **(D)** Representative histogram of ROS expression in CD45+ versus CD45^−^CECs from the spleen of a 15-day-old mouse. **(E)** Cumulative data showing mean fluorescence intensity (MFI) of ROS in CD45^+^/CD45^−^CECs of neonatal mice (day 15). **(F)** Representative plots of CD45 expression among splenic CECs versus their counterparts in the bone marrow (BM) of 15-day-old mice. **(G)** Cumulative data showing percentages of CD45+ CECs in the spleen versus bone marrow on neonatal mice. **(H)** Representative histogram of ROS expression in total CECs in the bone marrow compared to the spleen of a day-15 mouse. **(I)** Cumulative data showing mean fluorescence intensity (MFI) of ROS in the bone marrow versus spleen of neonatal mice.

To better characterize CECs in human infants, we analyzed the expression of different regulatory molecules on their surface (e.g. PDL-1/PDL-2, galectins, and CD73/CD39), however, unlike their counterparts in mice we observed negligible levels of these molecules (data not shown). Then to gain a better insight into the neonatal CECs, we analyzed the expression of CD45. We found that almost one-third of CECs in human infants express CD45 from birth to age 5 years (**Figures 2A, B**). Recently, we reported that cord blood CECs express NOX-2 gene and *via* the production of ROS enhance HIV-infection and replication in CD4^+^ T cells *in vitro* (31). Therefore, we decided to measure the production of ROS by CECs. Interestingly, we observed that CECs either from the human cord blood or peripheral blood of neonates expressed similar levels of ROS (**Figures 2C, D**). However, CD45^+^CECs appeared to be the major source of ROS compared to their CD45^−^ counterparts in human neonates (**Figures 2E–G**). Although we did not observe a significant difference in the frequency of CD45^+^CECs in human neonates during the development (**Figure 2B**), we found a substantial increase in the abundance of CD45^+^CECs after age 8-day in the spleen of neonatal mice which significantly increased at day 10 and remained constant until day 21, once again, the frequency of CD45^+^CECs significantly increased at day 25 (**Figures 3A, B**). Next, CECs from newborn mice were subjected to ROS production analysis to determine whether they have similar capabilities in terms of ROS production to human CECs. Interestingly, we found that CD45^+^CECs subpopulation in neonatal spleen were the most dominant source of ROS compared to their CD45^−^ siblings (**Figures 3C–E**). Moreover, we compared CD45+ expression in the bone marrow-derived CECs compared to their counterparts in the spleen. CECs from the bone marrow had significantly lower levels of CD45 compared to their counterparts in the spleen (**Figures 3F, G**). Subsequently, CECs from the bone marrow had lower ROS expression compared to the splenic CECs (**Figures 3H, I**). These observations indicate that CD45^+^CECs are to be the main source of ROS in both humans and mice. CD45, the receptor-like tyrosine phosphatase, is expressed on nucleated hematopoietic cells including erythroid progenitors. However, it gets downregulated as erythroid progenitors become mature (34). This implies that erythroid progenitors are the main source of ROS compared to their older siblings. Importantly, CECs in the bone marrow of mice express lower CD45 and have lower ROS production compared to those in the spleen.

### Human Neonatal CECs Have Immunosuppressive Properties and Suppress Both Monocytes and T Cells *In Vitro*

**Figure 4:**
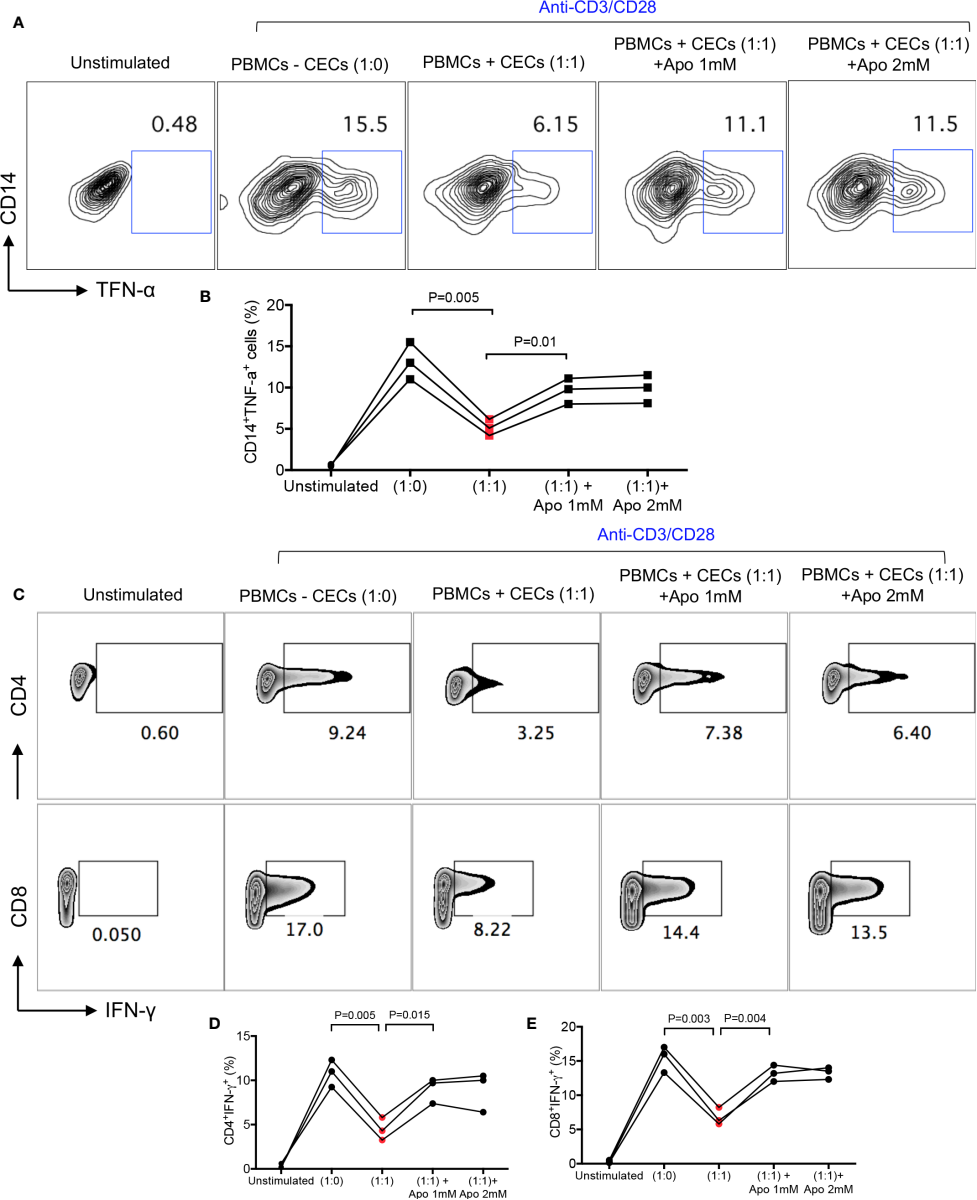
CECs from human infants exhibit immunosuppressive properties *in vitro*. **(A)** Representative plots, and **(B)** cumulative data showing TNF-α production by CD14+ cells following stimulation with LPS (2 μg/ml) in the absence/presence of CECs (1:1) ratio and presence of apocynin at shown concentrations for 6 h with a Golgi blocker. Samples are from three infants aged 2–4 weeks. **(C)** Representative plots, and **(D)** cumulative data showing IFN-γ production by CD4^+^ or **(E)** CD8^+^ T cells following stimulation with anti-CD3/CD28 in the absence/presence of CECs (1:1) ratio and presence of apocynin at shown concentrations for 18 h with an additional 6 h in the presence of a Golgi blocker. Samples are from three infants age 2 to 4 weeks.

We have previously shown that neonatal CECs in mice have immunosuppressive properties and suppress CD11b+ and CD11c+ cells (13, 14). In addition, we have reported that CECs suppress T cell activation and cytokine production *in vitro* and *in vivo* (13–15). This immunosuppression was mediated through the expression of arginase-II by CECs (13, 14). However, it was impossible to measure the arginase-II activity in human CECs. In contrast to mice, human neonatal CECs get lysed when exposed to the fixed/perm buffer for the intracellular staining. Despite this technical issue, we have already reported that human CECs express arginase-II mRNA (17). Thus, we believe human CECs similar to their counterparts in mice also express arginase-II. Since human CECs express NOX-2 gene and utilize ROS for their immunomodulatory functions (31), we decided to determine whether neonatal CECs vis ROS exhibit immunosuppressive properties. Therefore, neonatal PBMCs were cultured in the presence or absence of CECs and stimulated with LPS (2 μg/ml) and a Golgi blocker for 6 h. We found that CECs at 1:1 ratio significantly reduced TNF-α production by monocytes (CD14+ cells). These observations suggest that human neonatal CECs utilize ROS for their immunosuppressive properties as apocynin partially abrogated their inhibitory properties (**Figures 4A, B**). Similar effects were observed for IFN-γ production by both CD4^+^ and CD8^+^ T cells when stimulated with anti-CD3/CD28 antibodies in the presence/absence of CECs and a ROS scavenger (apocynin) (**Figures 4C–E**). Although we have shown that apocynin at 1 mM completely abrogated CECs-mediated enhanced HIV-1 infection/replication in CD4^+^ T cells in the cord blood (31), it partially reversed the immunosuppressive properties of neonatal CECs even when 2 mM apocynin was used (**Figures 4A–E**). This suggests that human CECs may utilize another potential mechanism to mediate their immunosuppressive properties (e.g. arginase-II). As such, the removal of CECs unleashed cytokine production by both monocytes and T cells in PBMCs of human neonates which supports their immunosuppressive properties.

### Neonatal CD45^+^CECs in Humans and Mice Express High Levels of VISTA

We have previously shown that CECs in the spleen of neonatal mice express substantial levels of VISTA, which *via* TGF-β promote the induction of Tregs from naïve CD4^+^ T cells (25). However, in that study, we reported that the cord blood CECs expressed a negligible amount of surface VISTA (25). In agreement with our previous studies, we confirmed that CECs in the spleen of neonatal mice express VISTA (**Supplementary Figure 2A**). In contrast to CECs in the human cord blood, we found that CECs in the neonatal peripheral blood express significant levels of VISTA (**Supplementary Figure 2B**). More importantly, these observations confirmed that VISTA was mainly expressed by CD45^+^CECs but not their CD45^−^ siblings (**Supplementary Figure 2C and 2D**). These data provide additional insight into the phenotypical characterization of neonatal CECs in human. Whether VISTA on human CECs plays a regulatory role or promotes the induction of Tregs merits further investigations.

### Pre-Emptive Depletion of CECs Protects Neonatal Mice Against Systemic Infection

**Figure 5:**
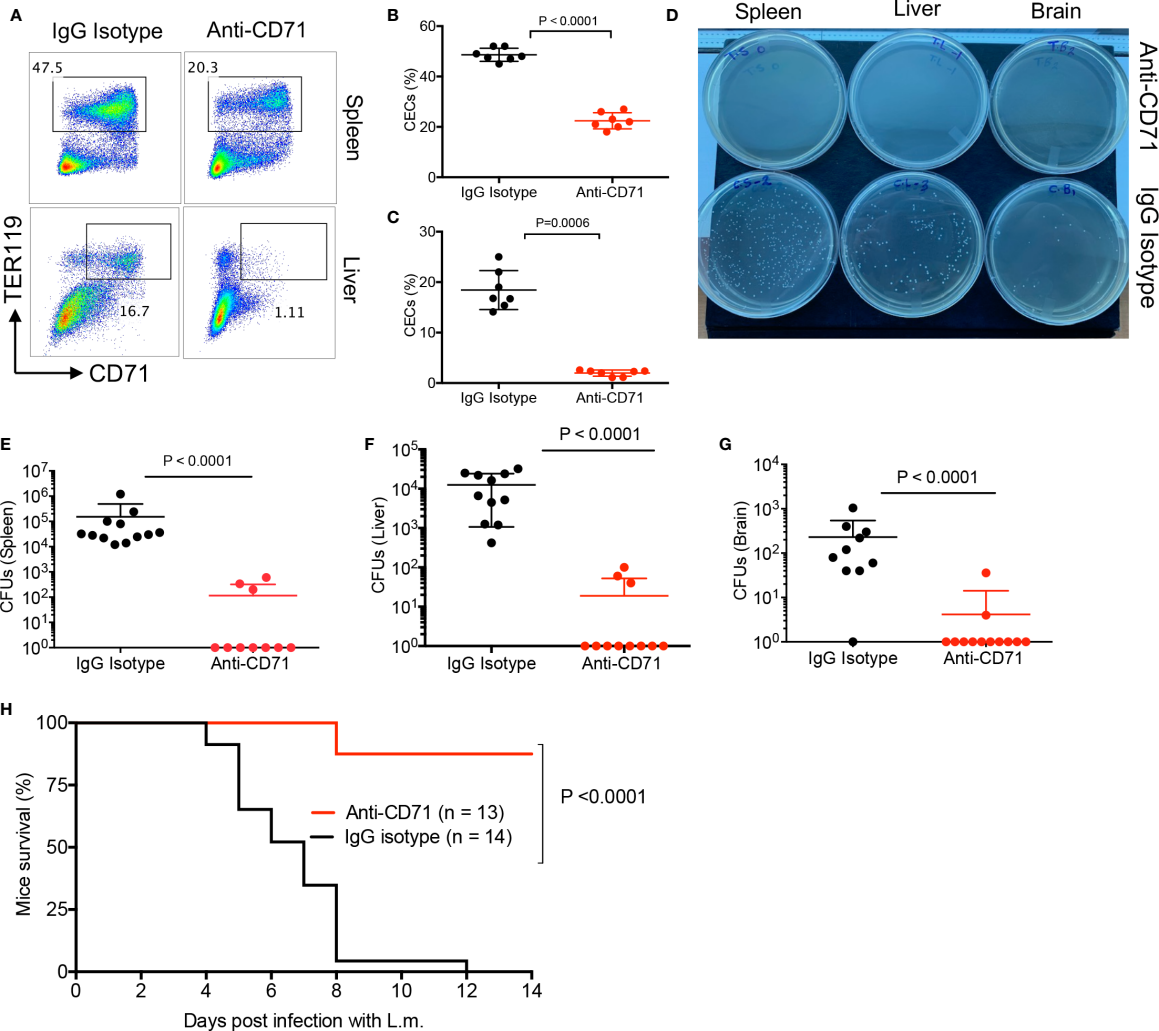
The depletion of CECs reduces bacterial load in tissues. **(A)** Representative plots and **(B)** and **(C)** cumulative data showing quantification of CECs in the spleen and liver of mice following anti-CD71 or IgG2a isotype control administration (i.p.) at days 4 and 5, measured 2 days later. **(D)** Representative L.B plates comparing bacterial colonies in anti-CD71 treated versus IgG2a isotype control treated mice. **(E)** Cumulative data of *Listeria monocytogenes* colony forming units (CFUs) in the spleen, **(F)** liver, and **(G)** brain of mice either treated with anti-CD71 antibody or IgG2a isotype control. **(H)** Survival for neonate mice either treated with isotype control or anti-CD71 at days 4 and 5 then orally administered with 1 × 10^7^ CFUs *Listeria monocytogenes* at day 6, and monitored over 2 weeks. Differences in mean values analyzed by two-tailed Student’s t test; p value is shown; N > 7/group, from at least two experiments.

We have previously shown that depletion of CECs resulted in increased protection against L.m. infection (13), however, in previous studies mice were infected *via* i.p. inoculation of bacteria. In the present study, to mimic the natural route of infection, we orally administered the bacteria to the neonatal mice. To determine whether the physiological abundance of CECs predisposes infants to systemic infection; first, we analyzed the effects of anti-CD71 neutralizing antibody (80 μg) on the frequency of CECs in the spleen and liver of treated mice versus controls. We observed this strategy significantly decreased the frequency of CECs in the spleen and liver of neonatal mice (**Figures 5A–C**). It is worth noting that as reported elsewhere (13), different anti-CD71 clones were used for the treatment (clone 8D3) versus flow cytometry staining (R17217 and C2F2). We administered the anti-CD71 antibody at day 4 and 5 followed by L.m infection (orally) at day 6. On day 8 (2 days post-infection), we quantified the number of bacterial colonies in the spleen, liver, and brain of neonatal treated mice compared to controls (isotype control). We observed that the depletion of CECs resulted in a significant decline in the number of L.m in the spleen, liver, and brain of neonatal mice (**Figures 5D–G**), which improved their survival rate (**Figure 5H**). Taken together, our data show that pre-emptive depletion of CECs significantly enhances the resistance of neonatal mice to L.m infection.

### Depletion of CECs Enhances the Recruitment of Immune Cells in the Spleen and Liver

**Figure 6:**
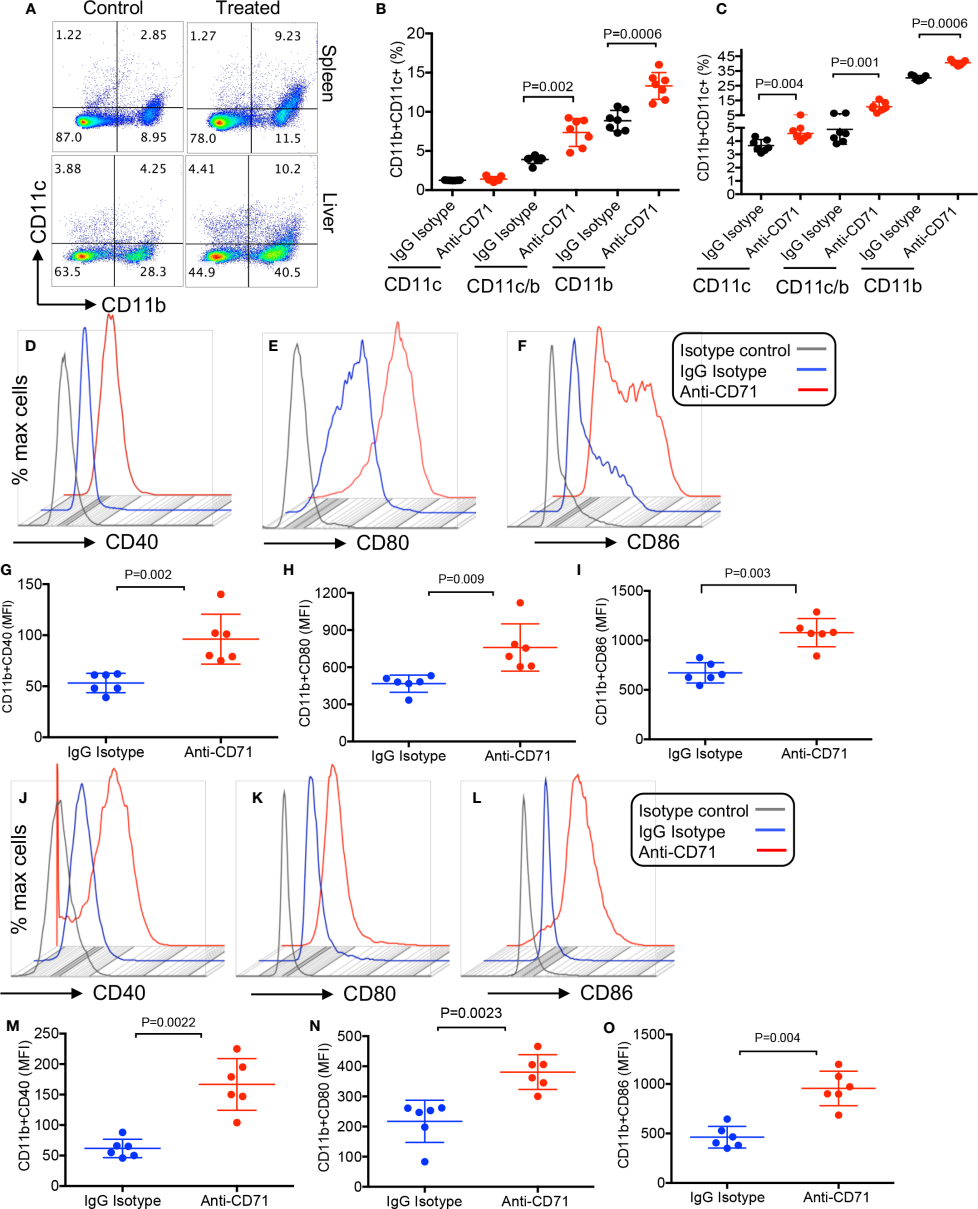
Increase of effector immune cells 48 h post the depletion of CECs and 24 h after oral infection with *Listeria monocytogenes.* **(A)** Representative plots and **(B)** quantification of CD11b+, CD11c+, and CD11b+CD11c+ cells in the spleen and **(C)** the liver. **(D–F)** Representative plots, and **(G–I)** quantification of activation markers CD40, CD80, and CD86 in splenic CD11b+ by mean fluorescence intensity (MFI). **(J–L)** Representative plots, and **(M–O)** quantification of activation markers CD40, CD80, and CD86 in hepatic CD11b+ by mean fluorescence intensity (MFI). Differences in mean values analyzed by two-tailed Student’s t test; p value is shown; N ≥ 6/group, from at least two experiments.

Considering the immunosuppressive properties of CECs (8, 13, 22, 24), we quantified the percentages of different immune cells after L.m oral infection. At 24 h after oral infection with L.m, depletion of CECs allowed for a surge in CD11b and CD11c cells in the spleen and liver of neonatal mice (**Figures 6A–C**). Importantly, these expanded CD11b/CD11c cells had an activated phenotype compared to those from the control group. This was evident by significantly higher expression of CD40, CD80, and CD86 levels on CD11b cells in the spleen (**Figures 6D–I**), and the liver (**Figures 6J–O**). A similar phenotype was observed for CD11c cells (data not shown). In addition, we found that depletion of CECs resulted in the recruitment of T cells in the spleen and liver of treated animals (**Supplementary Figures 3A–D**). Since T cells following activation/infection can upregulate CD71 (the transferrin receptor), we measured the surface expression of CD71 on splenic T cells. We observed although T cells is neonatal mice express about 10–15% CD71, the anti-CD71 antibody treatment did not have a significant effect on the expression of this molecule on both CD4^+^ and CD8^+^ T cells (**Supplementary Figures 3E, F**) despite its neutralizing effects on CECs (**Figure 5A**). These observations support a potential role for CECs in suppressing splenic and hepatic immune cells in the neonatal period.

## Discussion

In the present study, we investigated the frequency of CECs in the peripheral blood of human newborns from 1-day to 5 years of age. We found that CECs were highly abundant in the peripheral blood of human newborns at 1–7 days followed by 8–28 day but their frequency significantly declined afterward. This trend was opposite to what we observed in the spleen of neonatal mice, where CECs appeared to be significantly lower at days 3 and 5 but reached their maximum level by day 6–9. Based on our previous observations, CECs are a heterogeneous population of erythroid progenitors and precursors (22, 24). Therefore, we decided to phenotypically characterize human neonatal CECs by measuring the surface expression of PD-L, PDL-2, galectins (1, 3, and 9), and the ectoenzymes CD73/CD39. However, human neonatal CECs expressed none or a negligible amount of these molecules. To further characterize these cells, we investigated the expression of CD45, a transmembrane molecule shown on the membrane of all nucleated hematopoietic cells and their precursors (35). Erythrocytes do not express CD45 (32), however, they are generated from CD45+ hematopoietic stem cells (HSC) and downstream erythroid progenitors through cytokine signaling such as EPO and stem cell factor (33). Therefore, we analyzed the percentages of CD45^+^CECs among PBMCs in human newborns/children in comparison with their counterparts in the spleen of neonatal mice. Interestingly, we found that the majority of human CECs lacked CD45 but about one-third of them had the surface expression of CD45 without substantial changes throughout childhood. In contrast, the expression of CD45 on the surface of splenic CECs significantly increased as mice aged. Another interesting observation was that CECs from the bone marrow had significantly lower CD45 expression compared to their counterparts in the spleen of neonatal mice. This may suggest the differential properties of extramedullary generated CECs versus those produced in the medullary spaces. To establish the association of CD45 with the functionality of CECs, we analyzed ROS expression in CD45^+^CECs and CD45^−^CECs. In agreement with a report on the predominant expression of ROS in CD45^+^CECs compared to their CD45^−^ siblings in an animal tumor model (23), we observed that CD45^+^CECs consistently expressed higher levels of ROS in comparison to their CD45^−^ counterparts. It is worth noting that splenic CECs had significantly higher levels of ROS compared to their mates in the bone marrow. This could be explained by a higher proportion of CD45^+^CECs in the spleen versus the bone marrow. Higher numbers of CD45^+^CECs indicates the abundance of progenitors versus precursors in the spleen of neonatal mice (34). Recently, we reported that CECs from the human cord blood/placenta, and likewise CECs in the PBMCs of HIV-infected patients and anemic individuals express ROS (31). Specifically, we found that CECs from the human cord blood had substantial levels of NOX2 mRNA, while the other NOX paralogous (NOX 1, 3, 4, 5, DUOX1, and 2) were undetectable (31). Although the endogenous ROS generation by RBCs has been documented (36), CECs had significantly higher ROS production capacity compared to their mature counterparts. More importantly, we showed CECs release mitochondrial ROS, which its function can be abrogated by ROS-scavenger Apocynin but not by N-acetyl cysteine (31).

Previously, we have reported that CECs in neonatal mice express arginase-II and this enzymatic activity was required for their immunosuppressive properties (13, 14). In particular, CECs *via* depletion of arginine in the microenvironment suppress immune cell activation (13) and impair phagocytosis of CD11b^+^ cells *in vitro* (14). Similar observations have been made for other suppressor cells associated with tumor progression or persistent infection (37, 38). Nevertheless, measuring arginase-II activity in human CECs was technically impossible as they get lysed when exposed to the fixation/perming buffer. In contrast, mice CECs can be permed and stained intracellularly for arginase-II and any other intracellular molecules such as cytokines (e.g. TGF-β) (25). Despite this obstacle, we have been able to detect mRNA of arginase-II in human CECs from the cord blood (17, 31) but performing such studies on neonatal CECs was impossible because of the limited sample size.

We have previously shown that CECs in neonatal mice exhibit immunosuppressive properties *in vitro*, and their depletion *in vivo* was associated with an early increased in the recruitment and influx of protective immune cells (e.g. Ly6G neutrophils, CD11b^+^, CD11c^+^, and NK cells) into the lungs in a model of pertussis (14). This early recruitment and/or activation of innate immune cells into the lungs of anti-CD71 treated mice more likely contributed to the clearance of bacteria and protection against *Bordetella pertussis* infection in neonatal mice (14). Likewise, we found that CECs populate the spleen and liver of neonatal mice and compromise their innate immune system against the pre-natal pathogen *L. monocytogenes*. Listeriosis is 18 times more common in pregnant than non-pregnant women and is responsible for 6% of all sepsis and 4% of meningitis presenting in the first 48 h of life (39, 40). We found that the deletion of CECs triggered a surge in the presence of immune effector cells in the spleen and liver of neonatal mice. This initial response was accompanied by a subsequent decline in the tissue bacterial load. The lower bacterial load in the periphery of treated mice with the anti-CD71 antibody may be the reason for a fewer L.m in the brain of animals. These observations support the notion of immunosuppressive properties of CECs which renders neonates more susceptible to infection. This was supported by significantly lower bacterial load in different tissues (e.g. the spleen, liver and brain) and prolonged survival of neonatal mice when CECs were partially depleted. The immunosuppressive properties of CECs goes beyond the innate immunity as they hinder systemic/mucosal cellular and humoral responses against infection (15). Despite the extreme limitations in the volume of blood specimens from human newborns/infants, we were able to perform some basic studies to evaluate the immunological properties of human neonatal CECs. Our observations proved that CECs from the PBMCs of human infants suppress the production of TNF-α by monocytes and IFN-γ by T cells *in vitro*. Interestingly, these immunosuppressive effects were partially reversed in the presence of apocynin, a ROS scavenger. Although these observations provide a novel mechanistic role for these physiologically abundant CECs in human infants, partial abrogation of their immunosuppressive functions suggest the presence of another factor(s). Therefore, we believe that CECs in human infants may utilize other immunomodulatory molecules such as arginase-II to mediate their immunosuppressive actions. VISTA could be another potential inhibitory molecule utilized by CECs either *via* interaction with its ligand or through the promotion of Tregs (25).

Taken together, we have demonstrated that CECs are abundant in human newborns. They have immunosuppressive capabilities and therefore may contribute to the compromised innate immune response to pathogens in newborns. In addition, it is possible to speculate that their abundance at the time of neonatal immunization programs may interfere with the adaptive immune responses to vaccination.

More importantly, our findings provide additional support to the notion of active immune suppression in the neonatal period. Thus, the impaired immune response seen in newborns could be explained, in part, by the abundance of CECs and other suppressor cells (e.g. myeloid-derived suppressor cells (MDSCs) (41) to dampen robust immune responses to external (pathogens) or internal antigens (e.g. microbiome). Our observations with recent discoveries in the field (42), support the concept that the neonatal immunity is not under-developed but instead tightly regulated, smart, highly dynamic, and complex. Therefore, such a highly regulated immune system due to the presence of immunosuppressive cells (e.g. CECs and MDSCs) in newborns/infants may explain one potential reason for the mainly asymptomatic COVID-19 infection in this age group (43–49). Compelling evidence indicated that innate immune hyperactivation in driving the acute disease in SARS-CoV-2–infected adults (50). It makes sense to suggest that the differential immune components in the young may prevent excessive and potentially damaging immune responses to COVID-19 infection. Similarly, trained innate immunity and the abundance of long-lived MDSCs in children (51) diminishes any excessive inflammatory response to pathogens such as SARS-CoV-2 (52). Thus, the biased immune tolerance than resistance strategy (53) in newborns/infants might be protective against COVID-19 infection (54). A deeper understanding of immune components and mechanistic pathways responsible for the immune regulation in newborn is required for an effective therapeutic approach to promote their health.

We can acknowledge several limitations in this study a) our sample size might appear to be small for some age groups but obtaining blood from human newborns was extremely challenging and most parents were not willing to participate in the study. b) the other limitation was the blood volume, performing functional studies on 0.5–1 ml blood was almost impossible. We have been fortunate to collect a few ml of blood from some infants for performing those functional studies. c) Due to the ethical issues, we were unable to obtain the sex/exact age of donors, thus, some variation might be related to these factors. Based on our observations we believe CECs are higher in day-1 compared to day-7 and putting 1- to 7-day-old newborns in one group might explain some of the observed variations in the frequency of CECs.

## Data Availability Statement

The original contributions presented in the study are included in the article/**Supplementary Material**. Further inquiries can be directed to the corresponding author.

## Ethics Statement

The studies involving human participants were reviewed and approved by Human Review Ethics Boards at the University of Alberta approved human studies with the ethics # Pro0046080 and Pro00063463. All study participants gave written informed consent to participate in this study. Studies related to human newborns were mainly performed in Mexico. The Ethics Committee of the Hospital de la Mujer (Women´s Hospital), the Mexican Ministry of Health approved the study (Reg. HM-INV/2018:02.09). In addition, some neonatal blood specimens were collected at the University of Alberta Hospital from infants who had elective operations. The appropriate Institutional Human Review Ethics Boards at the University of Alberta approved such studies (ethics # Pro00001408). Parents gave written informed consent form to participate in the neonatal related studies in Mexico and Canada. Written informed consent to participate in this study was provided by the participants’ legal guardian/next of kin. The animal study was reviewed and approved by the Animal ethics board of the University of Alberta in strict accordance with the recommendations in the Guide for the Care and Use of Laboratory Animals of the Canadian Council for Animal Care with animal ethics # AUP00001021.

## Author Contributions

MV-L organized sample collection and processing, gave technical and logistic support and guidance, supervised data collection, analyses and reviewed the manuscript. VH-M and CR-E collected samples and performed the immunological studies. JM-R contributed in blood sample collection from children in Mexico. LW and BM assisted in recruitment of study subjects, sample collection in Edmonton and provided insight. OO performed the animal studies. SE conceived the research, supervised all of the study, assisted and performed some of the immunological assays, analyzed the data and wrote the manuscript. All authors contributed to the article and approved the submitted version.

## Funding

This work was supported by a New Investigator Award in Maternal and Child Health from the Canadian Institutes of Health Research (CIHR), Stollery Children’s Hospital Foundation/Women and Children Health Research Institute (WCHRI) and a Foundation grant from CIHR (all to SE). During this project VH-M received a MSc scholarship from CONACyT-Mexico.

## Conflict of Interest

The authors declare that the research was conducted in the absence of any commercial or financial relationships that could be construed as a potential conflict of interest.
