# Supplementary material for: CD71+ Erythroid Cells in Human Neonates Exhibit Immunosuppressive Properties and Compromise Immune Response Against Systemic Infection in Neonatal Mice
Source: Front Immunol. 2020 Nov 24;11:597433. doi: 10.3389/fimmu.2020.597433 (PMC7732591; doi:10.3389/fimmu.2020.597433)
Supplement: Supplementary file 1 [file DataSheet_1.pdf]

Supplementary Figure 1

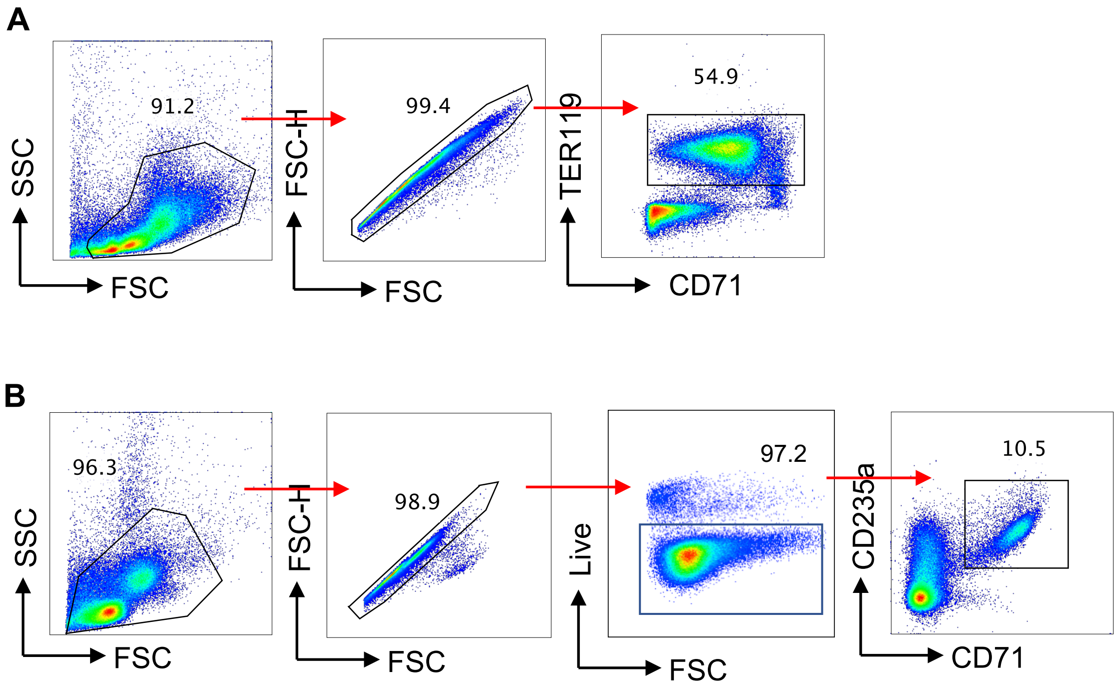

A) Representative flow cytometry plots showing the gating strategy for CD71+TER119+ erythroid cells in neonatal mice.

B) Representative flow cytometry plots showing the gating strategy for CD71+CD235a+ erythroid cells in human newborns.

Supplementary Figure 2

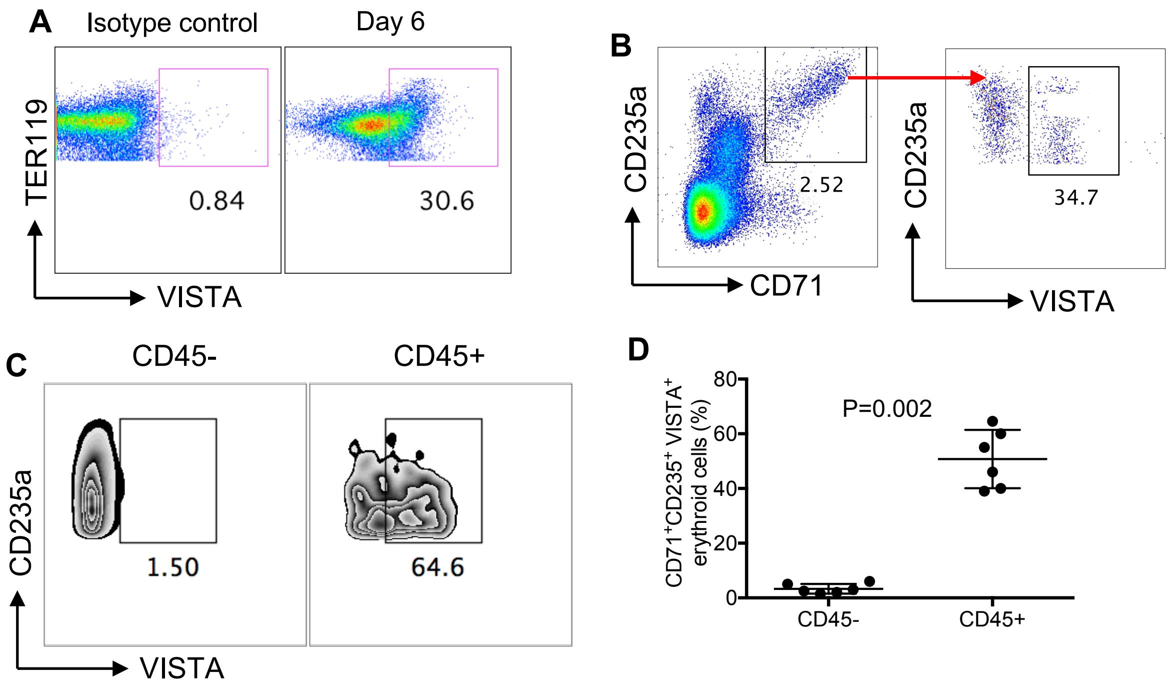

A) Representative plot showing expression of VISTA on mice CECs. **B**) Representative plots showing expression of VISTA on total CECs in a human newborn. **C**) Representative plots showing expression of VISTA on $CD45^-$ versus $CD45^+$ CECs in a human infant. **D**) Cumulative data showing percentages of VISTA expressing CECs among $CD45^-$ versus $CD45^+$ subpopulations in human infants.

Supplementary Figure 3

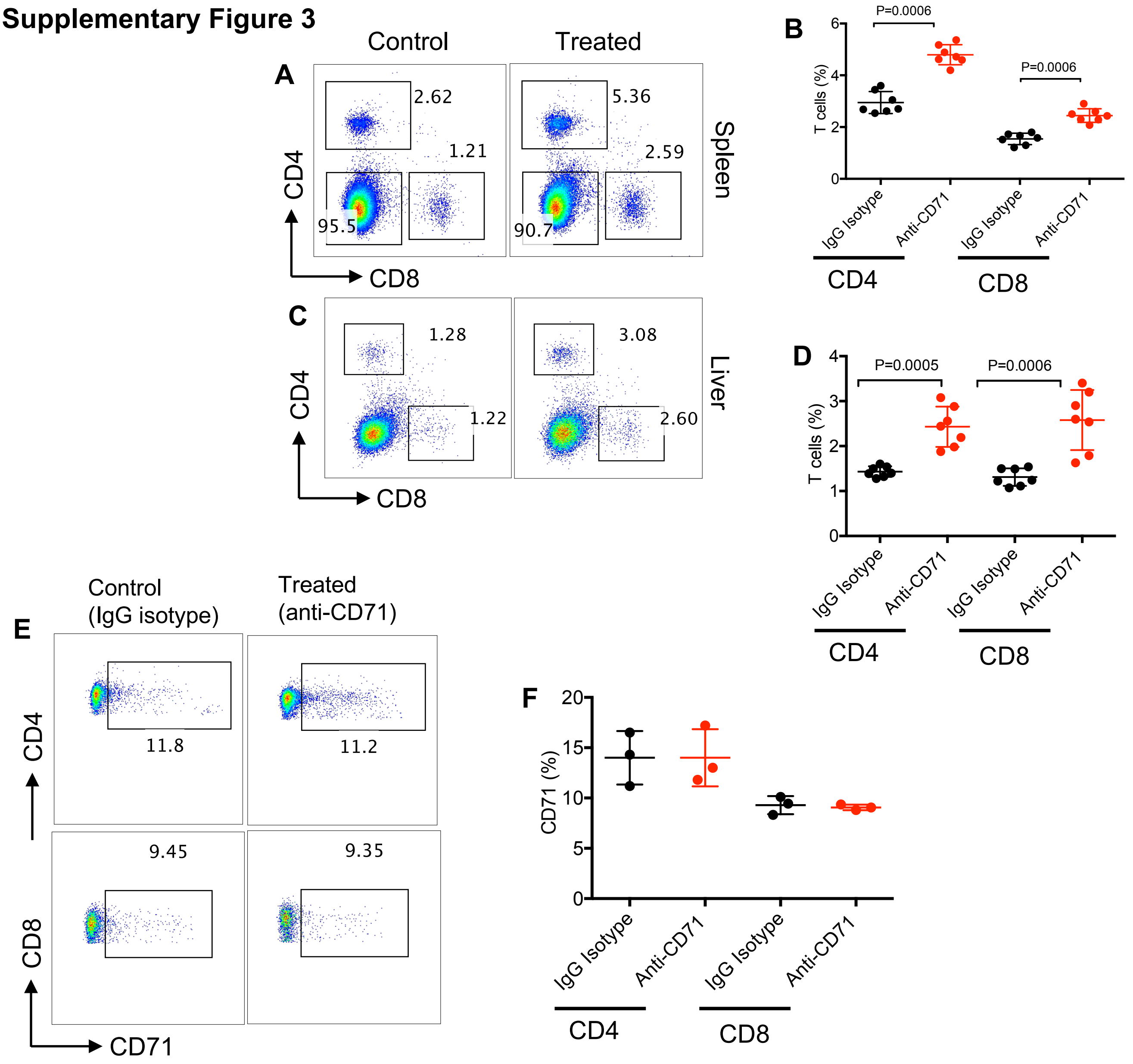

A) Representative flow cytometry plots, and (**B**) quantification of CD4⁺ and CD8⁺ T cells in the spleen of anti-CD71 treated versus controls. **C**) Representative flow cytometry plots, and (**D**) quantification of CD4⁺ and CD8⁺ T cells in the liver of anti-CD71 treated versus controls. Differences in mean values analyzed by 2-tailed student's t test; p value is shown; N=7/group, from at least 2 experiments. (**E**) Representative flow cytometry plots, and (**F**) cumulative data of CD71 expression on CD4+ or CD8+ T cells in IgG Isotype control treated versus anti-CD71 treated 6 days old neonatal mice. A representative experiment from two.
